# Natural History of Myocardial α_v_β_3_ Integrin Expression After Acute Myocardial Infarction: Correlation with Changes in Myocardial Blood Flow

**DOI:** 10.2967/jnumed.124.267514

**Published:** 2024-07

**Authors:** Matthieu Dietz, Christel H. Kamani, Colin Bousige, Vincent Dunet, Judith Delage, Vladimir Rubimbura, Marie Nicod Lalonde, Giorgio Treglia, Niklaus Schaefer, Wail Nammas, Antti Saraste, Juhani Knuuti, Nathan Mewton, John O. Prior

**Affiliations:** 1 Nuclear Medicine and Molecular Imaging Department, Lausanne University Hospital, Lausanne, Switzerland;; 2 Institut de Cardiologie des Hospices Civils de Lyon, CarMeN Laboratory INSERM 1060, Université Claude Bernard Lyon 1, Lyon, France;; 3 Service de Médecine Nucléaire, Hospices Civils de Lyon, Lyon, France;; 4 Department of Cardiology, University Hospital of Lausanne, Lausanne, Switzerland;; 5 Laboratoire des Multimatériaux et Interfaces, UMR CNRS 5615, Université Claude Bernard Lyon 1, Villeurbanne, France;; 6 University of Lausanne, Lausanne, Switzerland;; 7 Radiopharmacy Unit, Department of Pharmacy, University Hospital of Lausanne, Lausanne, Switzerland;; 8 Department of Cardiology, Ensemble Hospitalier de la Côte, Morges, Switzerland;; 9 Imaging Institute of Southern Switzerland, Ente Ospedaliero Cantonale, Bellinzona, Switzerland;; 10 Faculty of Biomedical Sciences, Università della Svizzera Italiana, Lugano, Switzerland;; 11 Heart Center, Turku University Hospital, Turku, Finland; and; 12 Turku PET Centre, Turku University Hospital and University of Turku, Turku, Finland

**Keywords:** integrin α_v_β_3_, PET, myocardial infarction, angiogenesis, RGD

## Abstract

Angiogenesis is an essential part of the cardiac repair process after myocardial infarction, but its spatiotemporal dynamics remain to be fully deciphered.^68^Ga-NODAGA-Arg-Gly-Asp (RGD) is a PET tracer targeting α_v_β_3_ integrin expression, which is a marker of angiogenesis. **Methods:** In this prospective single-center trial, we aimed to monitor angiogenesis through myocardial integrin α_v_β_3_ expression in 20 patients with ST-segment elevation myocardial infarction (STEMI). In addition, the correlations between the expression levels of myocardial α_v_β_3_ integrin and the subsequent changes in ^82^Rb PET/CT parameters, including rest and stress myocardial blood flow (MBF), myocardial flow reserve (MFR), and wall motion abnormalities, were assessed. The patients underwent ^68^Ga-NODAGA-RGD PET/CT and rest and stress ^82^Rb-PET/CT at 1 wk, 1 mo, and 3 mo after STEMI. To assess ^68^Ga-NODAGA-RGD uptake, the summed rest ^82^Rb and ^68^Ga-NODAGA-RGD images were coregistered, and segmental SUVs were calculated (RGD SUV). **Results:** At 1 wk after STEMI, 19 participants (95%) presented increased ^68^Ga-NODAGA-RGD uptake in the infarcted myocardium. Seventeen participants completed the full imaging series. The values of the RGD SUV in the infarcted myocardium were stable 1 mo after STEMI (1 wk vs. 1 mo, 1.47 g/mL [interquartile range (IQR), 1.37–1.64 g/mL] vs. 1.47 g/mL [IQR, 1.30–1.66 g/mL]; *P* = 0.9), followed by a significant partial decrease at 3 mo (1.32 g/mL [IQR, 1.12–1.71 g/mL]; *P* = 0.011 vs. 1 wk and 0.018 vs. 1 mo). In segment-based analysis, positive correlations were found between RGD SUV at 1 wk and the subsequent changes in stress MBF (Spearman ρ: *r* = 0.17, *P* = 0.0033) and MFR (Spearman ρ: *r* = 0.31, *P* < 0.0001) at 1 mo. A negative correlation was found between RGD SUV at 1 wk and the subsequent changes in wall motion abnormalities at 3 mo (Spearman ρ: *r* = *–*0.12, *P* = 0.035). **Conclusion:** The present study found that α_v_β_3_ integrin expression is significantly increased in the infarcted myocardium 1 wk after STEMI. This expression remains stable after 1 mo and partially decreases after 3 mo. Initial α_v_β_3_ integrin expression at 1 wk is significantly weakly correlated with subsequent improvements in stress MBF, MFR, and wall motion analysis.

## Visual Abstract

**Figure:**
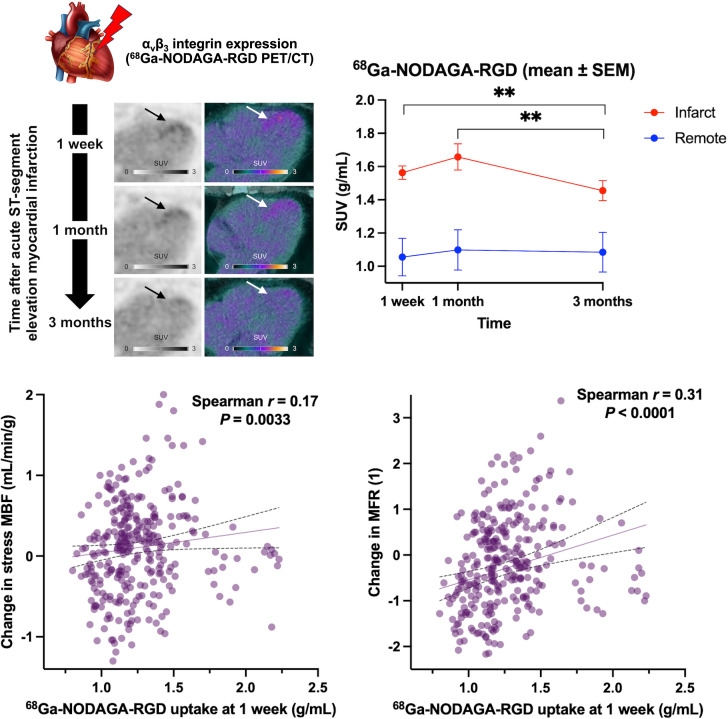

The initiation of cardiac repair after a myocardial infarction (MI) requires a complex series of processes. In the first few days after reperfusion, an inflammatory phase with intense inflammation and immune cell infiltration enables the infarct to be cleared of damaged cells. This phase is followed by a reparative and proliferative phase over the next several days, with a believed peak around day 7; it includes the resolution of inflammation, cardiac fibroblast proliferation, scar formation, and angiogenesis, which are essential parts of the repair process (1*,*^2^). However, the spatiotemporal dynamics of angiogenesis after an MI remain to be fully deciphered (2). To prevent heart failure after MI, an improved understanding of myocardial angiogenesis is essential for the future development of effective and targeted treatments.

The transmembrane glycoprotein α_v_β_3_ integrin is involved in cell interaction with the extracellular matrix, migration, and proliferation. Integrin α_v_β_3_ is expressed on activated endothelial cells, where it plays a critical role in the angiogenic process within the myocardium after injury (3*–*6). Expression of integrin α_v_β_3_ has also been reported in other various cell types, such as fibroblasts and activated macrophages (7*–*10). This diversity in expression may introduce some confounding effects. However, α_v_β_3_ imaging holds promise for assessment of cardiac wound healing and repair after MI (11*,*^12^). In 2015, in a publication summarizing advanced techniques to evaluate angiogenesis, the American Heart Association cited the use of radiotracers to assess integrin expression (13). ^68^Ga-NODAGA-Arg-Gly-Asp (RGD) is a PET tracer having a high binding affinity for α_v_β_3_ and favorable biokinetics (14*,*^15^). A widely used PET perfusion tracer in clinical practice is ^82^Rb, allowing for accurate measurement of rest and stress myocardial perfusion in absolute units, with a test–retest methodologic precision of around 20% (16).

We hypothesized that myocardial ^68^Ga-NODAGA-RGD uptake is increased during the peak of the proliferative phase after acute ST-segment elevation MI (STEMI) and is reduced during later stages of infarct healing. The objective of the present study was to assess the expression levels of myocardial α_v_β_3_ integrin at 1 wk after STEMI and their potential evolution at 1 and 3 mo. In addition, the correlations between the expression levels of myocardial α_v_β_3_ integrin and the subsequent changes in ^82^Rb-PET/CT parameters, including rest and stress myocardial blood flow (MBF), myocardial flow reserve (MFR), and wall motion abnormalities, were assessed.

## MATERIALS AND METHODS

### Study Design

This was a prospective single-center trial conducted in the Centre Hospitalier Universitaire Vaudois (Lausanne, Switzerland). All participants were included within the first 12 h after symptom onset and underwent reperfusion (supplemental materials; available at http://jnm.snmjournals.org). The study was approved by the ethics committee of the canton of Vaud (protocol CER-VD 429/14) and registered at ClinicalTrials.gov (NCT03809689). All patients gave written informed consent.

### PET/CT Imaging

**FIGURE 1.**
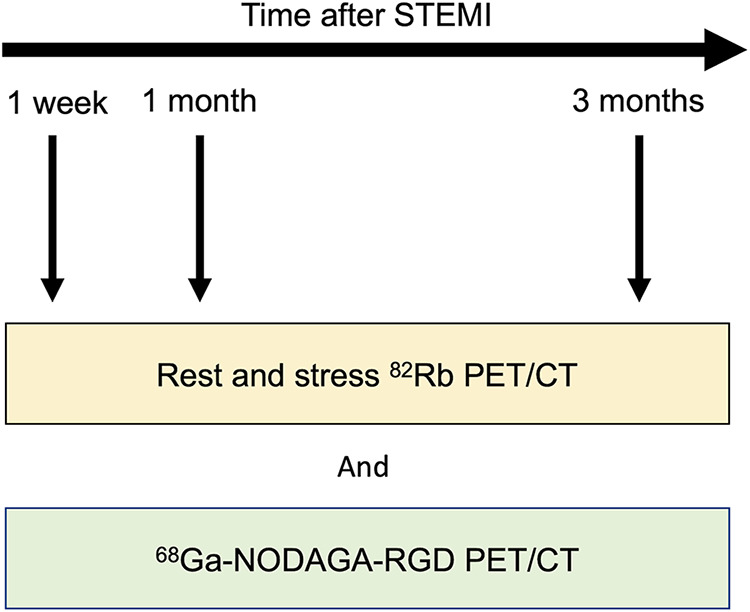
Experimental timeline.

All participants underwent ^68^Ga-NODAGA-RGD PET/CT and rest and stress ^82^Rb-PET/CT at 1 wk (4–10 d) after STEMI. Then, the participants underwent repeated ^68^Ga-NODAGA-RGD PET/CT and rest and stress ^82^Rb-PET/CT at 1 and 3 mo after STEMI (Fig. 1). The ^82^Rb-PET/CT imaging was performed as previously described (17), with the complete imaging acquisition protocol reported in the supplemental materials. Briefly, a 5 MBq/kg dose of ^82^Rb (Ruby-Fill generator and ^82^Rb elution system; Jubilant DraxImage) was administered through an automated infusion system for 15–25 s, and 3-dimensional dynamic PET images were acquired for 6.1 min (12 × 8, 5 × 12, 1 × 30, 1 × 60, and 1 × 120 s). Stress acquisitions were then performed using the same protocol. Then, a median of 159 MBq of ^68^Ga-NODAGA-RGD was injected as an intravenous bolus and was followed by a PET acquisition of 10 min after a median of 60 min. Images were reconstructed using ordered-subsets expectation maximization algorithms.

### PET Image Analysis

Image analyses were performed using PMOD (version 4.3; PMOD Technologies).

#### ^82^Rb-PET/CT Analysis

Perfusion was assessed quantitatively, measuring MBF in mL/min/g at rest and stress using a 1-tissue-compartment model with a flow-dependent extraction correction (supplemental materials). Perfusion was also assessed visually and semiquantitatively. A segmental wall motion analysis was performed on rest-gated ^82^Rb-PET images using a 5-point scoring scale, and the summed rest score for wall motion was derived. Rest left ventricular end-diastolic and end-systolic volumes, as well as ejection fraction, were computed.

#### ^68^Ga-NODAGA-RGD PET/CT Analysis

^68^Ga-NODAGA-RGD images were assessed visually and quantitatively. To assess the RGD uptake, the summed rest ^82^Rb images and the ^68^Ga-NODAGA-RGD images were coregistered (Supplemental Fig. 1). Polar maps of ^68^Ga-NODAGA-RGD uptake expressed as SUVs (SUV; measured activity concentration [Bq/mL] × body weight [g]/injected dose at the time of image decay correction [Bq]) were generated, and mean segmental SUVs were calculated (RGD SUV). The characterization of the infarcted myocardium and the remote myocardium is detailed in the supplemental materials.

### Statistical Analysis

No statistical methods were used to predetermine sample size. All statistical analyses were performed using R software (version 4.2.2, R Project). A *P* value of less than 0.05 was considered statistically significant. Continuous variables were expressed as median and interquartile range (IQR) or as mean ± SEM. The comparisons were performed using a Wilcoxon signed-ranks test. Categoric data were expressed as count and percentage. The Friedman test was used to compare continuous variables across multiple time points. When statistical significance was reached, post hoc Dunn multiple-comparison tests were used for pairwise comparisons. The correlations between RGD SUV and the subsequent changes in rest MBF, stress MBF, MFR, and wall motion analysis were assessed on all segments using the Spearman correlation analysis. The tests were selected because of the nonnormal distribution of the data, as determined by the Shapiro–Wilk test.

## RESULTS

### Participants

In total, 20 participants were included in the study (20% female; age, 63 y [IQR, 58–69 y]; Supplemental Table 1). Among the participants, 19 (95%) had no previous history of cardiovascular disease before the onset of acute STEMI. Only one participant had a history of prior coronary revascularization, with percutaneous coronary intervention (PCI) and coronary artery bypass graft surgery.

PCI was performed on all the participants 3.8 h (IQR, 2.3–6.3 h) after symptoms onset. Invasive angiography found single-vessel obstructive disease in 7 (35%) participants and multivessel obstructive coronary artery disease in 13 (65%). All participants underwent stenting using drug-eluting stents of the culprit lesion. One participant (5%) had unsuccessful reperfusion with a post-PCI thrombolysis in MI flow grade 1.

The 20 participants underwent rest and stress ^82^Rb-PET/CT and ^68^Ga-NODAGA-RGD PET/CT imaging 9 d (IQR, 7–10 d) after STEMI. A total of 18 participants underwent a second ^82^Rb-PET/CT and ^68^Ga-NODAGA-RGD PET/CT study 32 d (IQR, 30–37 d) after STEMI, and 17 participants underwent a third ^82^Rb-PET/CT and ^68^Ga-NODAGA-RGD PET/CT study 89 d (IQR, 83–96 d) after STEMI and completed the full imaging series. In segment-based analysis, rest and stress MBF, MFR, wall motion analysis, and RGD SUV were assessed for the full imaging series in 289 segments (17 × 17 participants).

### Imaging Findings 1 Week After STEMI

Complete reperfusion, as indicated by absence of a significant perfusion defect (summed stress score < 4), was observed in 6 participants (33%). The rest MBF was 0.50 mL/min/g (IQR, 0.42–0.58 mL/min/g) across the entire myocardium, 0.58 mL/min/g (IQR, 0.48–0.60 mL/min/g) in the remote myocardium, and 0.38 mL/min/g (IQR, 0.34–0.52 mL/min/g) in the infarcted myocardium. Rest left ventricular ejection fraction was less than 50% in 12 participants (60%). A total of 14 participants (70%) demonstrated segmental wall motion abnormalities (summed rest score for wall motion > 0).

A total of 19 participants (95%) presented RGD-positive segments (*n* = 66); those segments were always included in myocardial tissue perfused by the culprit coronary artery. Among those 66 positive segments, 55 were obtained from the 17 participants who completed the full imaging series and were selected to represent the infarcted myocardium. Among the 6 participants who experienced a complete reperfusion and had no significant ^82^Rb PET perfusion defect (summed stress score < 4), RGD-positive segments were detected in 5 (83%).

### Temporal Changes

**FIGURE 2.**
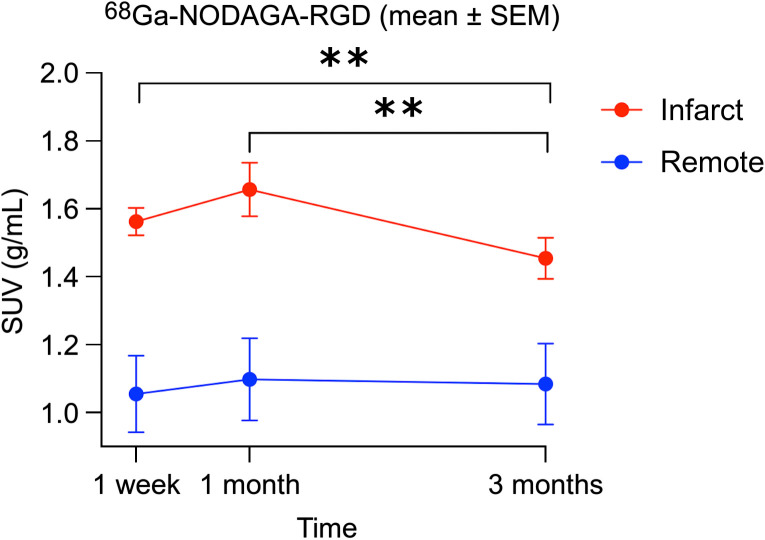
α_v_β_3_ integrin expression in infarcted myocardium remained stable 1 mo after STEMI, followed by significant partial decrease at 3 mo. **P ≤ 0.01.

**FIGURE 3.**
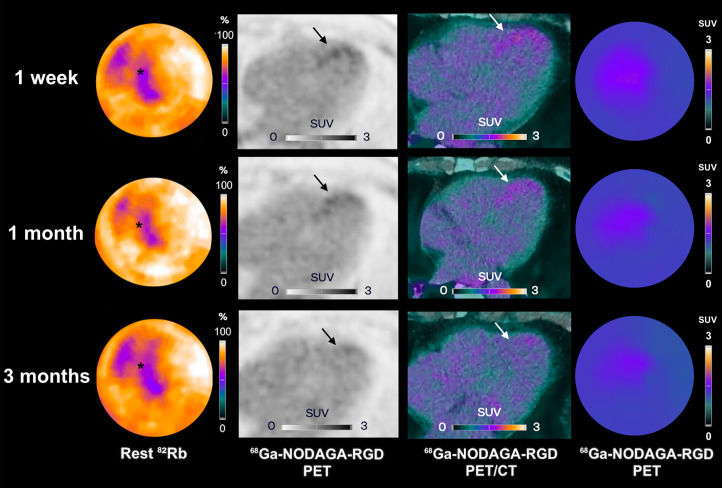
Participant example with decrease in α_v_β_3_ integrin expression levels (arrows) in infarcted myocardium (asterisks) after 1 wk to follow-up at 3 mo. Shown are rest ^82^Rb, ^68^Ga-NODAGA-RGD PET, and ^68^Ga-NODAGA-RGD PET/CT images at 1 wk (first row), 1 mo (second row), and 3 mo (third row) after STEMI.

The values of the RGD SUV in the infarcted myocardium were stable 1 mo after STEMI (*P* = 0.9 for post hoc comparison of 1 wk vs. 1 mo; Table 1), followed by a significant decrease at 3 mo (*P* = 0.011 for post hoc comparison with 1 wk, *P* = 0.018 for post hoc comparison with 1 mo; Table 1; Figs. 2 and 3). There was no significant change in RGD SUV in the remote myocardium throughout the follow-up (Friedman test *P* = 0.59). At all time points, the RGD SUVs of the infarcted myocardium and the remote myocardium were significantly different (all *P* < 0.001).

**TABLE 1.** Temporal Changes in Infarcted and Remote Myocardium

Parameter	1 wk	1 mo	3 mo
Infarcted myocardium			
RGD SUV (g/mL)	1.47 (1.37–1.64)	1.47 (1.30–1.66)	1.32 (1.12–1.71)*
Rest MBF (mL/min/g)	0.38 (0.34–0.52)	0.44 (0.31–0.58)	0.44 (0.37–0.52)
Stress MBF (mL/min/g)	1.16 (0.71–1.61)	1.49 (0.77–1.96)	1.51 (0.92–2.18)^†^
MFR (1)	3.04 (1.96–3.90)	2.94 (2.42–3.62)	3.50 (2.32–4.65)
Remote myocardium			
RGD SUV (g/mL)	1.05 (0.92–1.11)	1.01 (0.99–1.11)	1.05 (0.94–1.15)
Rest MBF (mL/min/g)	0.58 (0.48–0.60)	0.57 (0.46–0.75)	0.50 (0.49–0.69)
Stress MBF (mL/min/g)	1.88 (1.30–2.19)	1.99 (1.24–2.16)	1.92 (1.22–2.39)
MFR (1)	3.35 (2.67–4.16)	3.19 (2.28–3.51)	2.98 (2.27–4.75)

* *P* = 0.011 vs. 1 wk and *P* = 0.018 vs. 1 mo.

† *P* = 0.035 vs. 1 wk.

Variables are expressed as median and IQR. Number 1 after MFR indicates unitless dimension.

**FIGURE 4.**
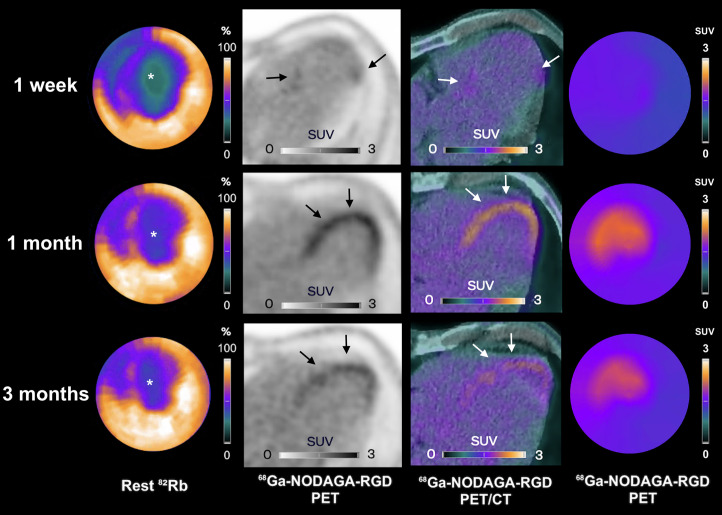
Participant example with increase in α_v_β_3_ integrin expression levels (arrows) throughout follow-up within infarcted myocardium (asterisks). Shown are rest ^82^Rb, ^68^Ga-NODAGA-RGD PET, and ^68^Ga-NODAGA-RGD PET/CT images at 1 wk (first row), 1 mo (second row), and 3 mo (third row) after STEMI. ^68^Ga-NODAGA-RGD uptake started in border zone at 1 mo and subsequently extended into necrotic infarct core at both 1 and 3 mo.

In participants with significant ^82^Rb-PET/CT perfusion defects, areas of positive RGD uptake matched the extent of perfusion defects, with some slight extensions in border zones. In the 2 participants with the most severe infarcts (rest MBF in the infarcted myocardium at 1 wk of 0.30 mL/min/g and 0.26 mL/min/g), the RGD SUVs of the infarcted myocardium increased by at least 20% at 1 mo, without a change in clinical status or occurrence of events (Fig. 4; Supplemental Fig. 2). In these 2 participants, the RGD uptake started in the border zone and extended into the necrotic infarct core (Fig. 4). The RGD uptake was relatively homogeneous in all the other participants with smaller infarcts.

In participant-based analysis, global ^82^Rb-PET/CT parameters and hemodynamics during ^82^Rb-PET/CT imaging did not significantly change between initial and follow-up imaging (Supplemental Tables 2 and 3). Left ventricular ejection fraction improved by at least 5% in 8 (12%) of the 17 participants who completed the full imaging series. In segment-based analysis, rest MBF improved by at least 20% in 163 segments (56%), stress MBF improved by at least 20% in 164 segments (57%), MFR improved by at least 20% in 151 segments (52%), and wall motion abnormalities improved (difference score for wall motion ≥ 1) in 61 segments (21%).

### Correlations Between RGD SUV and Subsequent Changes in ^82^Rb-PET Parameters

In segment-based analysis, positive correlations were found between initial RGD SUV at 1 wk and the subsequent changes at 3 mo in stress MBF (Spearman ρ: *r* = 0.13, *P* = 0.026) and MFR (Spearman ρ: *r* = 0.18, *P* = 0.003). There was no correlation between RGD SUV at 1 wk and the subsequent changes in rest MBF at 3 mo (Spearman ρ: *r* = *–*0.08, *P* = 0.19). A negative correlation was found between RGD SUV at 1 wk and the subsequent changes in wall motion abnormalities at 3 mo (Spearman ρ: *r* = *–*0.12, *P* = 0.035).

**FIGURE 5.**
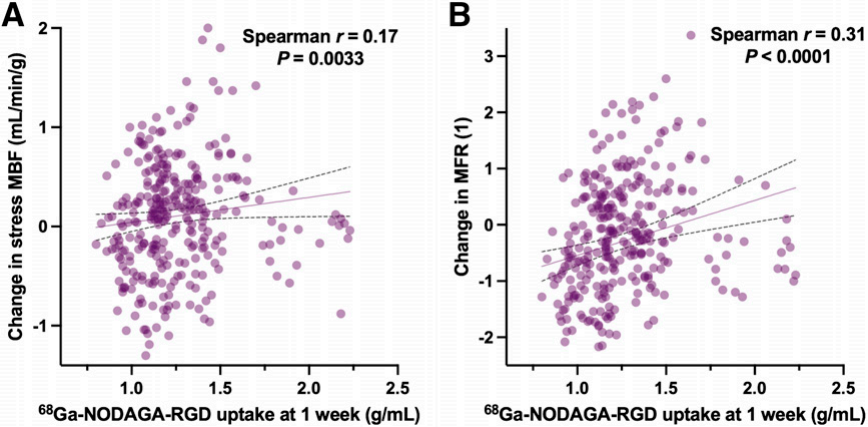
Correlations of ^68^Ga-NODAGA-RGD uptake at 1 wk after STEMI with subsequent changes in stress MBF (A) and MFR (B) at 1 mo. Data points represent mean segmental values.

In further analysis, positive significant correlations were found between RGD SUV and the subsequent changes in stress MBF and MFR in the 1-wk to 1-mo period (Spearman ρ: *r* = 0.17, *P* = 0.0033, and Spearman ρ: *r* = 0.31, *P* < 0.0001, respectively; Fig. 5). No correlation was found between RGD SUV at 1 mo and the subsequent changes at 3 mo in stress MBF (Spearman ρ: *r* = 0.05, *P* = 0.44) or in MFR (Spearman ρ: *r* = –0.02, *P* = 0.72). No correlation was found during these shorter periods between RGD SUV and the subsequent changes in wall motion abnormalities (1-wk to 1-mo period, Spearman ρ: *r* = 0.02, *P* = 0.7; 1-mo to 3-mo period, Spearman ρ: *r* = 0.06, *P* = 0.33).

## DISCUSSION

The present study reported in vivo, in humans, an increased α_v_β_3_ integrin expression in the infarcted myocardium 1 wk after STEMI. The levels of α_v_β_3_ integrin expression remained stable at 1 mo after STEMI and partially decreased at 3 mo, suggesting that angiogenesis remains active 3 mo after the infarct. Moreover, there were significant weak correlations between the segmental levels of α_v_β_3_ integrin expression at 1 wk and the subsequent improvements in stress MBF, MFR, and wall motion analysis.

The kinetics of angiogenesis after MI in humans remain poorly understood (2). The results reported here on myocardial α_v_β_3_ integrin expression in vivo in the human infarcted myocardium found persistent activity at 1 mo, followed by a partial decline at 3 mo. The present results are consistent with the serial changes in vascular endothelial growth factor levels observed in several studies within the peripheral blood of patients with acute MI. These levels were reported to peak at 6 wk and subsequently decline to baseline levels at 5 mo (18). Another study found a progressive increase in vascular endothelial growth factor levels that started on the first day after acute MI, peaked at 2 wk, and then subsequently declined (19). Hence, it can be thought that this prolonged increase is necessary to preserve the remaining myocardium and limit hypoxic cellular destruction.

The prolonged α_v_β_3_ integrin upregulation found here agrees with previous studies using radiolabeled RGD peptides after MI (4*,*^11^*,*^20^*,*^21^). In a rat model, the uptake of the ^18^F-galacto-RGD tracer was detected only 3 d after MI and reached its highest levels between 1 and 3 wk; the levels were still detectable after 6 mo. Moreover, the evolution of ^18^F-galacto-RGD tracer uptake correlated well with neovascularization as assessed by immunohistochemical CD31 staining (4). Similarly, the uptake pattern of ^18^F-AlF-NOTA-PRGD2, in the infarcted area at various time points after MI, also started after only 3 d and peaked between 1 and 3 wk; a subsequent partial decrease was reported 4 mo after MI (20). In humans, Jenkins et al. (11) found that ^18^F-labeled RGD uptake was increased within 2 wk in the infarcted myocardium. It persisted but was reduced after approximately 10 wk. There was no uptake found at sites of established old infarctions. It is of note that in the present study, an examination of established old infarction sites was not conducted since none of the participants had an MI history before acute STEMI. Nevertheless, in our trial evaluating tumoral angiogenesis (NCT02666547), which includes some patients with a prior history of MI, we do not observe any visually positive uptake of ^68^Ga-NODAGA-RGD in the myocardium.

The present study reported significant correlations between the segmental levels of α_v_β_3_ integrin at 1 wk and the subsequent improvements in clinically relevant physiologic parameters, such as stress MBF, MFR, and wall motion analysis. Although the correlations were weak, they were determined using the Spearman rank correlation coefficient, which is insensitive to outliers. Additionally, the alignment of PET findings could have been more accurate, since distinct radiotracers (with high-energy positrons) and acquisitions were used. A stronger effect of these correlations can be assumed, which means that the correlations are likely relevant. Notably, a slightly stronger correlation was observed between α_v_β_3_ integrin expression levels and improvements in MFR, which is considered a more robust independent prognostic factor than is stress MBF (22). Furthermore, no correlation was found between α_v_β_3_ integrin expression levels and subsequent changes in rest MBF, a parameter unrelated to clinical outcomes and unaffected by post-MI recovery (23*,*^24^). The significant but weak correlation between α_v_β_3_ integrin expression levels at 1 wk and functional outcomes aligns with previous challenges to translate microscopic levels of angiogenesis into functional improvements. For example, Wu et al. reported only trends toward lower perfusion defects and metabolism deficits in vascular endothelial growth factor–treated animal models, without statistically significant changes (25). Nevertheless, as here, several studies suggested that elevated α_v_β_3_ integrin expression after ischemic myocardial injury is associated with subsequent improvement in regional left ventricular function (6*,*^11^*,*^12^*,*^26^*,*^27^). Recently, using the same PET tracer with ^68^Ga-NODAGA-RGD, Nammas et al. reported in humans that α_v_β_3_ integrin expression levels 1 wk after MI were linked to regional and global systolic dysfunction, as well as elevated left ventricular filling pressure, and predicted improved global left ventricular function 6 mo after MI (12). Despite these insights, further studies are warranted to better understand whether the angiogenic response is associated with functional recovery.

Variability was observed in the temporal pattern of ^68^Ga-NODAGA-RGD uptake, with a notable increase found at 1 mo in 2 participants. Interestingly, the 2 participants affected were those experiencing the most severe infarcts. On the basis of these findings, it remains uncertain whether this increase in ^68^Ga-NODAGA-RGD uptake is related to intensified reendothelialization and angiogenesis or indicates a shift toward myofibroblast cell types, suggesting an intensified fibrotic response. This delayed phase of repair is characterized by a reduction in inflammation and angiogenesis and a reorganization of the extracellular matrix through myofibroblast-driven type I and III collagen production (1). This more intense uptake during follow-up in infarcted areas where perfusion is most profoundly reduced, with a trend toward a delayed 1-mo peak, may reflect the need for a more intense healing process in those tissues.

The present trial had several limitations. Inflammatory cells and fibroblasts can express integrins such as α_v_β_3_ (7*–*10), which can lead to reduced specificity. This may explain the weak correlations found between the 1-wk ^68^Ga-NODAGA-RGD uptake and the subsequent improvements in flow, as well as the absence of correlation found from 1 mo onward. The positron range is higher for ^68^Ga than for ^18^F, which may affect image quality, especially in relatively small moving structures. Nevertheless, ^68^Ga-NODAGA-RGD has the advantage of straightforward synthesis at room temperature with high radiochemical yield and purity. It can be radiolabeled rapidly (<30 min) and in a fully automated good-manufacturing-practice–compliant manner. Furthermore, the on-site availability of ^68^Ge/^68^Ga generators for centers without access to a cyclotron makes it a good alternative for ^18^F-labeled compounds. Moreover, a higher uptake of multimeric RGD tracers than of monomeric tracers was reported (28). A multimodal imaging strategy, such as PET/MRI, could precisely delineate the area at risk and its border zone. The study would have been strengthened by an independent measure of regional function using echocardiography or MRI. Furthermore, the contribution of nonspecific uptake to ^68^Ga-NODAGA-RGD accumulation cannot be excluded. However, positive uptake was found in segments with preserved perfusion and in segments with low perfusion, and as radiolabeled RGD-based peptides have shown rapid clearance from the circulation (4*,*^14^), it seems unlikely that nonspecific uptake related to changes in vascular permeability played a major role. Finally, the potential impact of standard medication regimens for coronary artery disease (e.g., statins, antihypertensives) on the uptake of ^82^Rb or ^68^Ga-NODAGA-RGD remains uncertain.

## CONCLUSION

The present study found that α_v_β_3_ integrin expression is significantly increased in the infarcted myocardium 1 wk after STEMI. This expression remained stable after 1 mo and partially decreased after 3 mo. Initial α_v_β_3_ integrin expression at 1 wk was significantly weakly correlated with subsequent improvements in stress MBF, MFR, and wall motion analysis. An enhanced comprehension of the mechanistic aspects of infarct α_v_β_3_ integrin expression could provide multiple therapeutic options.

## DISCLOSURE

The Swiss Heart Foundation (Bern, Switzerland) provided financial support in developing the ^68^Ga-RGD radiopharmaceutical. Matthieu Dietz was a PhD student partially supported by research fellowship awards from the Société Française de Radiologie, Paris, France, and from the Agence Régionale de Santé Auvergne-Rhone-Alpes, Lyon, France. Antti Saraste discloses grants from the Research Council of Finland and Finnish Foundation for Cardiovascular Research and speaker or consultancy fees from Abbott, AstraZeneca, BMS, Janssen, Novartis, and Pfizer, outside the submitted work. Juhani Knuuti received consultancy fees from GE Healthcare and Synektik Pharma and speaker fees from Bayer, Lundbeck, Boehringer Ingelheim, Pfizer, and Siemens, outside the submitted work. No other potential conflict of interest relevant to this article was reported.

## Notes

KEY POINTS **QUESTION:** What are the spatiotemporal dynamics of α_v_β_3_ integrin expression after MI, and its correlation with changes in MBF, using ^68^Ga-NODAGA-RGD PET/CT and rest and stress ^82^Rb-PET/CT at 1 wk, 1 mo, and 3 mo after STEMI? **PERTINENT FINDINGS:** This prospective clinical study showed that α_v_β_3_ integrin expression was significantly increased in the infarcted myocardium 1 wk after STEMI. This expression remained stable after 1 mo and partially decreased after 3 mo. Initial α_v_β_3_ integrin expression at 1 wk was significantly weakly correlated with subsequent improvements in stress MBF, MFR, and wall motion analysis. **IMPLICATIONS FOR PATIENT CARE:** An enhanced comprehension of the mechanistic aspects of infarct α_v_β_3_ integrin expression could provide multiple therapeutic options.
